# Case Report: Radiotherapy combined with DEB-TACE for hemorrhagic locally advanced solid tumors: a report of 3 cases

**DOI:** 10.3389/fonc.2025.1647251

**Published:** 2025-12-17

**Authors:** Yunfeng Guan, Bin Shen

**Affiliations:** 1 Huzhou Central Hospital, Affiliated Central Hospital of Huzhou University, Huzhou, China; 2 Huzhou Central Hospital, The Fifth School of Clinical Medicine of Zhejiang Chinese Medical University, Huzhou, Zhejiang, China

**Keywords:** complete response, DEB-TACE, locally advanced solid tumors, radiotherapy, tumor hemorrhage

## Abstract

For patients with certain types of unresectable locally advanced solid tumors, concurrent chemoradiotherapy (CCRT) is the standard treatment regimen. However, in clinical practice, specific patient populations such as the elderly, those with poor performance status (PS) scores, or those with significant underlying comorbidities often struggle to tolerate CCRT, resulting in unfavorable prognosis, and the clinical outcomes are even worse when accompanied by tumor bleeding. This paper reports three specific cases of locally advanced solid tumors (including one case each of lung cancer, esophageal cancer, and cervical cancer) with bleeding that could not tolerate concurrent chemotherapy. All cases received combined treatment of radiotherapy and drug-eluting bead transarterial chemoembolization (DEB-TACE). The results demonstrated that hemorrhagic symptoms were rapidly and effectively controlled in all patients, and all achieved long-term complete response (CR) after treatment. These findings suggest that the combination of radiotherapy and DEB-TACE may be a promising treatment strategy for such specific patients with hemorrhagic locally advanced solid tumors.

## Introduction

Concurrent chemoradiotherapy (CCRT) is the standard treatment for unresectable locally advanced solid tumors such as lung cancer, esophageal cancer, and cervical cancer (1–3). However, some patients cannot tolerate the toxicities associated with concurrent intravenous chemotherapy (e.g., myelosuppression, gastrointestinal reactions) and thus opt for sequential therapy or radiotherapy alone, compromising treatment efficacy (4). Additionally, tumor-related bleeding not only disrupts treatment but can also be life-threatening in severe cases (5, 6). Current clinical approaches for hemostasis include pharmacological agents, mechanical compression, and endoscopic interventions (7). Transarterial embolization (TAE), which selectively occludes tumor-feeding vessels, has demonstrated both reliable hemostatic efficacy and antitumor effects for various solid tumors (8).

Drug-eluting bead transarterial chemoembolization (DEB-TACE) is an advanced interventional technique evolved from conventional TAE. By replacing traditional embolic agents with drug-loaded microspheres, DEB-TACE preserves the hemostatic benefits of TAE while enhancing antitumor effects through sustained local chemotherapy release (9). Moreover, the localized chemotherapeutic delivery may radiosensitize tumor cells, creating synergistic therapeutic effects. For patients with bleeding, unresectable locally advanced solid tumors who are ineligible for intravenous chemotherapy, combining radiotherapy with DEB-TACE could represent a promising strategy.

This study reports three cases of locally advanced squamous cell carcinomas (one each of lung, esophageal, and cervical origin) with active tumor bleeding and intolerance to concurrent intravenous chemotherapy. All patients were managed with a combined modality approach involving radiotherapy and DEB-TACE.

## Case 1

**Figure 1:**
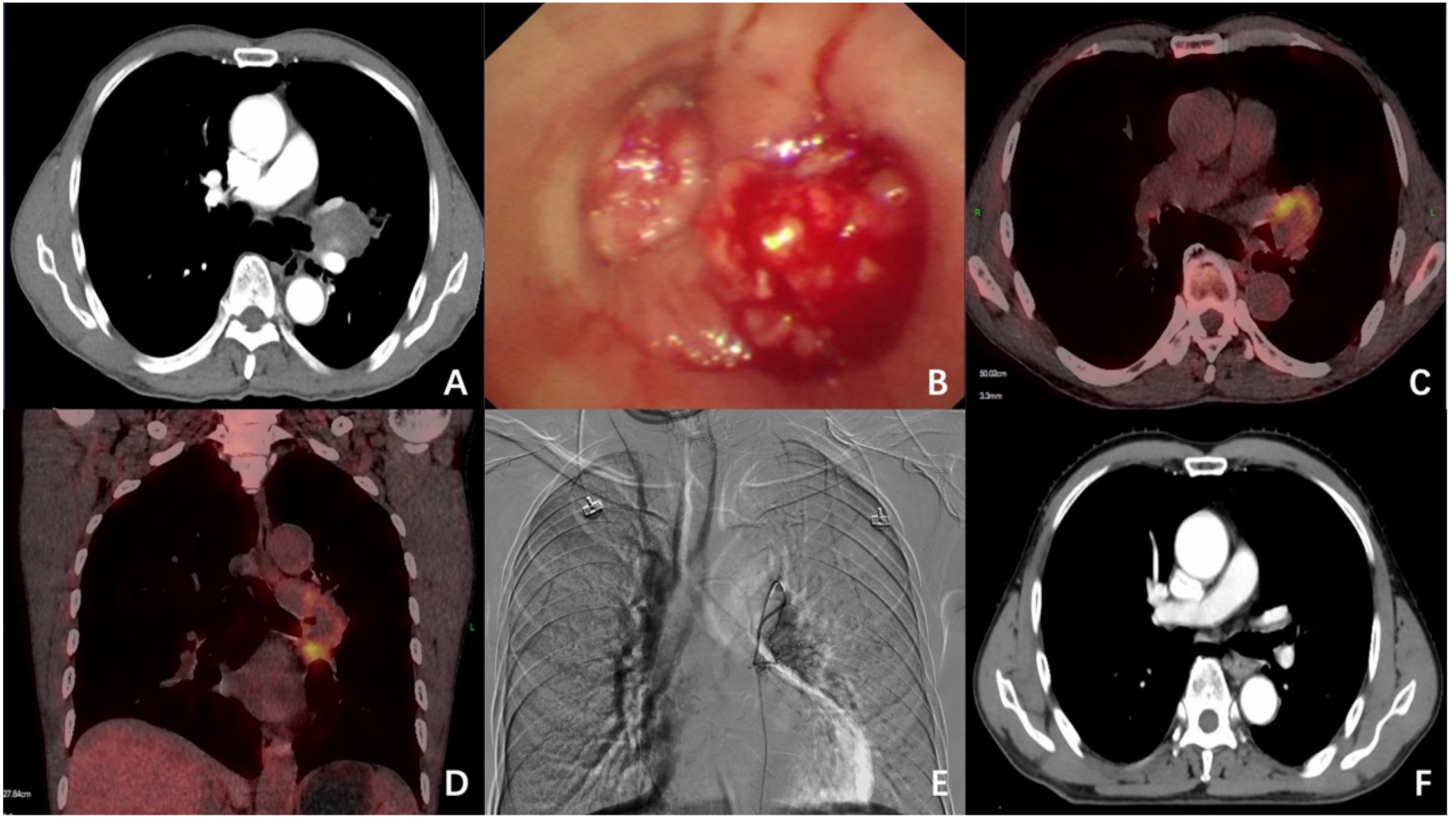
Imaging, endoscopic and DSA findings of Case 1: **(A)** Chest CT demonstrates a 4.1×5.2 cm mass at the left hilum. **(B)** Bronchoscopy reveals a neoplasm with hemorrhage obstructing the orifices of both left upper and lower lobe bronchi. **(C, D)** PET-CT shows markedly increased metabolic activity in the left hilar mass (SUVmax 8.3) and enlarged, hypermetabolic 10L lymph nodes (SUVmax 8.1). **(E)** DSA reveals hypertrophied bronchial arteries supplying the tumor, which exhibits rich vascularization. **(F)** Follow-up chest CT at 1-month post-combination therapy demonstrates complete tumor resolution.

A 60-year-old male with a history of megaloblastic anemia was admitted in May 2022 presenting with “intermittent hemoptysis accompanied by chest tightness for 2 months, aggravated over 1 week”. The patient reported approximately 30 mL of daily hemoptysis during the most recent week. Physical examination revealed a performance status (PS) score of 1 and diminished breath sounds in the left lung. Laboratory investigations demonstrated moderate anemia (hemoglobin 73 g/L) and elevated squamous cell carcinoma antigen (SCC 2.6 ng/mL).Diagnostic imaging included a chest CT scan showing a 4.1×5.2 cm irregular soft tissue mass at the left hilum with associated bronchial stenosis (**Figure 1A**). Bronchoscopy demonstrated a neoplasm with active bleeding at both the left upper and lower lobe bronchial orifices (**Figure 1B**), with histopathological confirmation of squamous cell carcinoma. Subsequent PET-CT imaging revealed markedly increased metabolic activity in the primary lesion (maximum standardized uptake value [SUVmax] 8.3) and metastatic involvement of the 10L lymph node group (SUVmax 8.1) (**Figures 1C, D**), establishing a clinical stage of cT3N1M0 (Stage IIIA).

Given the patient’s moderate anemia and active hemoptysis, he was deemed unable to tolerate the additional toxicity of concurrent intravenous chemotherapy. DEB-TACE was selected as the initial intervention due to its dual capability for rapid hemostasis and localized anti-tumor effect. This treatment strategy, including the rationale for DEB-TACE, was thoroughly discussed with the patient, and written informed consent was obtained prior to the procedure. Digital subtraction angiography (DSA) during the procedure clearly demonstrated hypertrophic and tortuous tumor-feeding arteries with intense tumor staining (**Figure 1E**). The intervention utilized HepaSphere^®^ microspheres (50-100μm), where irinotecan (100 mg) was slowly loaded over 30 minutes. The microspheres were then slowly infused until achieving complete blood flow stasis in the tumor vasculature. Following a 3-day interval after DEB-TACE, the patient initiated volumetric modulated arc therapy (VMAT) with a definitive radiation dose of 60 Gy administered in 30 fractions.

The therapeutic response was notable for immediate cessation of hemoptysis within 24 hours post-embolization, followed by significant improvement in hemoglobin levels within one week. Follow-up computed tomography performed 1 month after completing radiotherapy demonstrated complete radiological response (**Figure 1F**). The patient subsequently received 24 months of maintenance therapy with durvalumab. At the most recent follow-up evaluation (31 months post-treatment), the patient maintained a durable complete response without evidence of disease progression. Treatment-related toxicities were limited to grade 1 esophagitis and grade 2 myelosuppression according to Common Terminology Criteria for Adverse Events (CTCAE) version 5.0 criteria, both of which were effectively managed with appropriate supportive measures.

## Case 2

**Figure 2:**
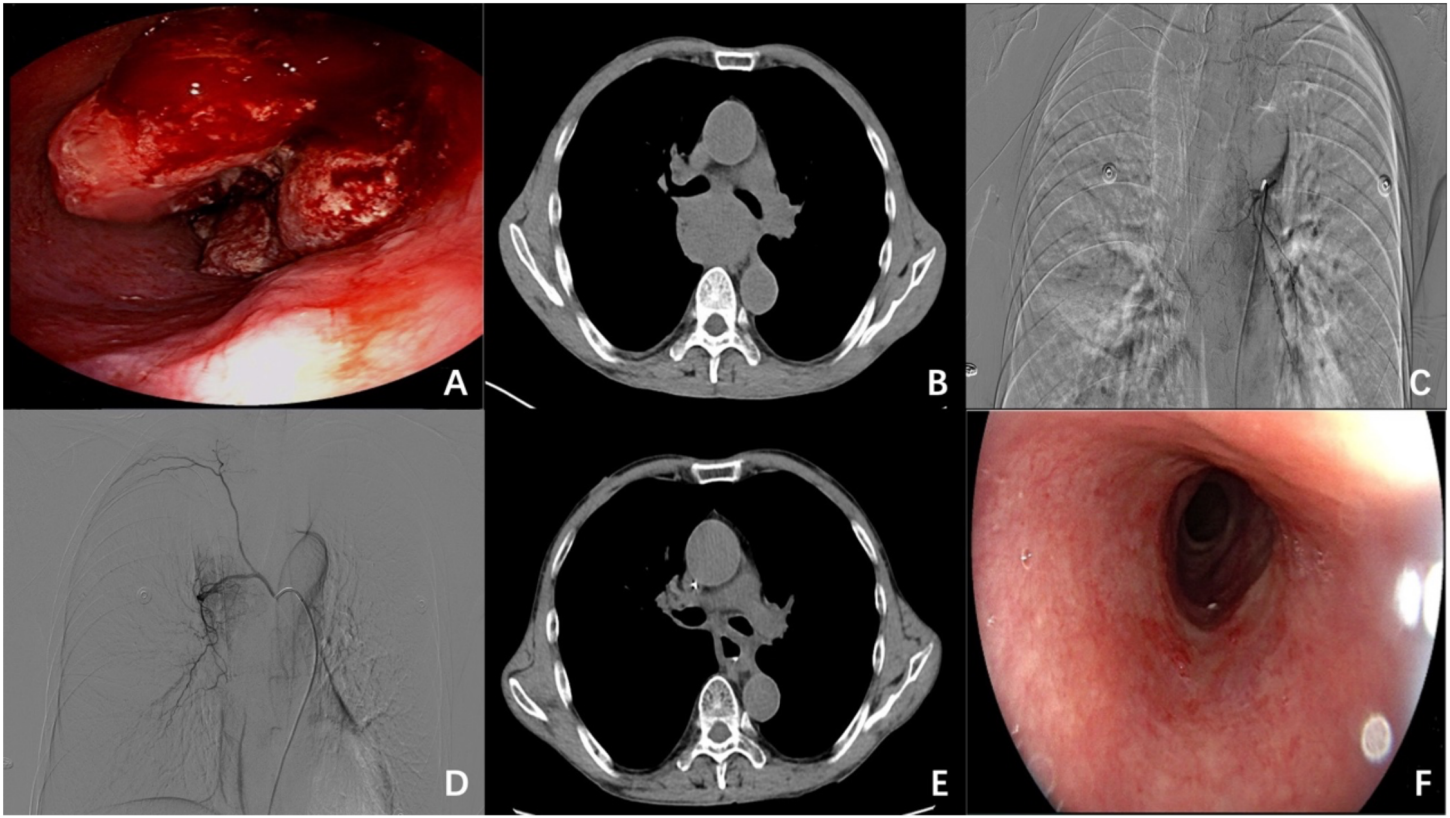
Imaging, endoscopic and DSA findings of Case 2: **(A)** Gastroscopy demonstrates a neoplasm causing significant luminal stenosis with active bleeding at 28–35 cm from the incisors. **(B)** Chest CT reveals marked wall thickening in the mid-esophagus. **(C, D)** DSA shows tumor blood supply from esophageal branches of bilateral bronchial arteries with prominent tumor vascularity. **(E)** Follow-up chest CT at 1-month post-combination therapy shows complete resolution of the primary tumor. **(F)** Post-treatment gastroscopy at 3 months displays radiation-induced mucosal changes without residual tumor.

A 69-year-old male presented with progressive dysphagia and melena for 1 month and was admitted in June 2023. Initial assessment revealed poor nutritional status (BMI 17.9 kg/m²), grade 3 dysphagia, and PS 2. Laboratory tests demonstrated moderate anemia (Hb 88 g/L) with persistent occult blood in stool. Gastroscopy identified an esophageal neoplasm causing significant luminal stenosis at 28–35 cm from incisors (**Figure 2A**), with biopsy confirming SCC. CT showed a mid-esophageal tumor without transmural invasion or metastatic lymphadenopathy (**Figure 2B**), clinically staged as cT3N0M0 (stage II).

Owing to the patient’s poor nutritional status, anemia, and active bleeding, CCRT was considered high-risk. Therefore, the multidisciplinary team opted for a combined approach with DEB-TACE and radiotherapy. The treatment plan was explained to the patient, and informed consent was obtained for the DEB-TACE procedure and subsequent radiotherapy. Angiography revealed tumor blood supply from esophageal branches of bilateral bronchial arteries (**Figures 2C, D**). DEB-TACE was performed using identical drug-loading protocol as Case 1. Radiotherapy (54Gy/30 fractions) commenced 3 days post-procedure.

Clinical outcomes included complete resolution of melena by day 5 and significant dysphagia improvement. Follow-up CT at 1 month and endoscopy at 3 months confirmed complete response (**Figures 2E, F**). During 17 months of follow-up, the patient maintained complete remission without recurrence. Treatment-related toxicities included grade 2 radiation esophagitis and grade 2 leukopenia, both of which were effectively managed with standard supportive measures.

## Case 3

**Figure 3:**
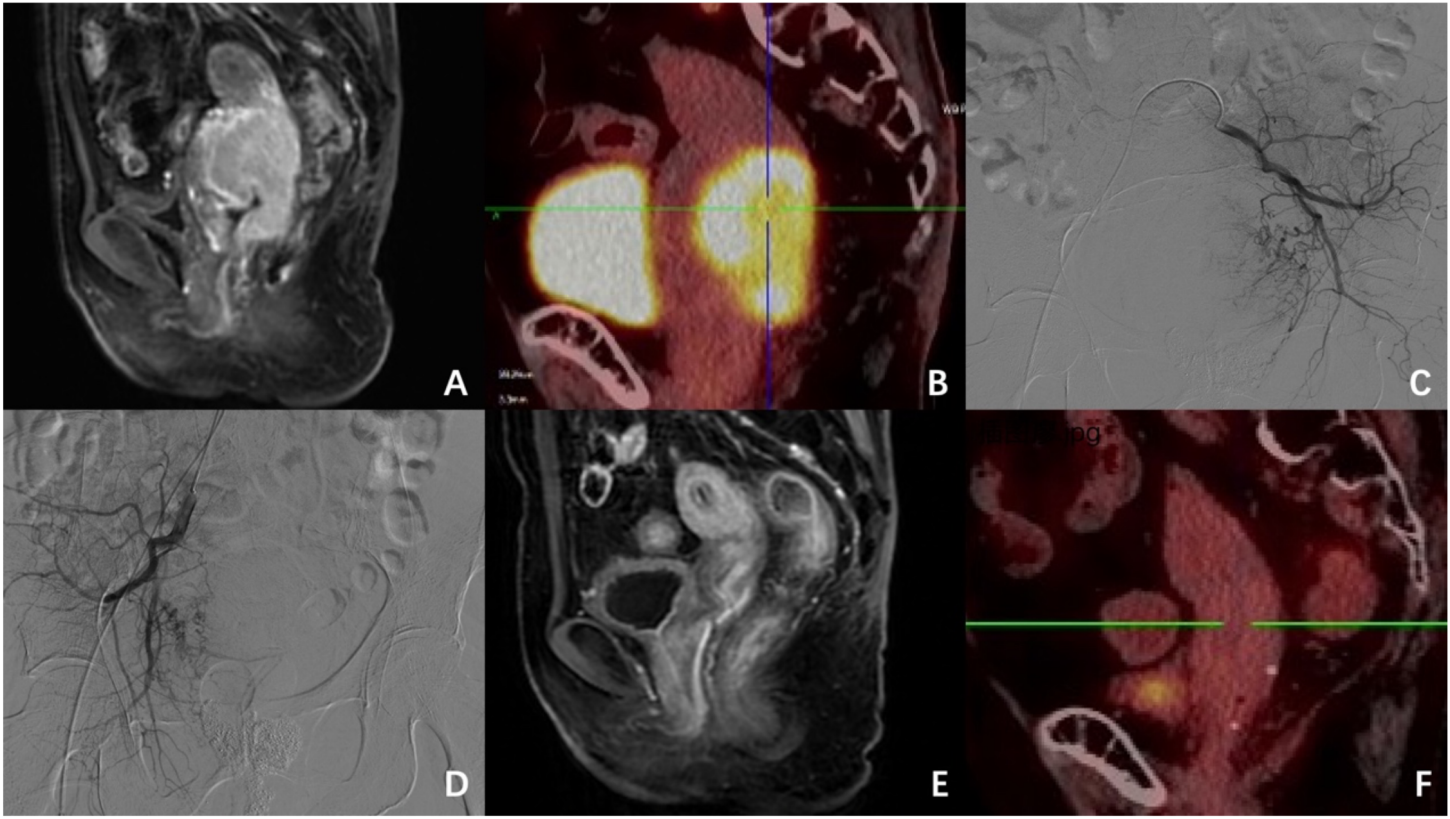
Imaging and DSA findings of Case 3: **(A)** Pelvic MRI demonstrates a 6.5×4.5 cm heterogeneously enhancing cervical mass with posterior extension to the rectal serosa. **(B)** PET-CT reveals markedly increased metabolic activity (SUVmax 14.2) in the cervical lesion. **(C, D)** Bilateral uterine artery angiography shows hypervascular tumor supply with intense neovascularization. **(E, F)** Follow-up MRI and PET-CT at 1-month post-combination therapy confirm complete resolution of the primary lesion without residual metabolic activity.

A 72-year-old postmenopausal female presented to our institution in February 2022 with a chief complaint of irregular vaginal bleeding persisting for three months. A gynecological examination revealed a markedly enlarged, indurated cervix with spontaneous bleeding, showing tumor infiltration involving the posterior vaginal fornix. Laboratory investigations demonstrated moderate anemia (hemoglobin 64 g/L) and elevated squamous cell carcinoma antigen levels (SCC 5.5 ng/mL). Diagnostic imaging included a pelvic MRI which identified a 6.5×4.5 cm cervical mass exhibiting contrast enhancement with posterior extension to the rectal serosal layer (**Figure 3A**). Subsequent PET-CT imaging revealed markedly increased metabolic activity in the cervical lesion (SUVmax 14.2) without evidence of extrapelvic metastatic spread (**Figure 3B**). Histopathological examination of biopsy specimens confirmed the diagnosis of squamous cell carcinoma, classified as FIGO stage IIIB.

During preoperative preparation for radiotherapy, the patient developed refractory vaginal hemorrhage that was unresponsive to conventional packing measures. This was accompanied by a progressive decline in hemoglobin levels to a nadir of 56 g/L, necessitating transfusion of packed red blood cells. This critical situation precluded the safe administration of CCRT. Following an urgent multidisciplinary consultation, DEB-TACE was identified as the appropriate intervention to achieve immediate hemostasis and initiate local tumor control. The procedure’s risks, benefits, and alternatives were thoroughly discussed with the patient, from whom informed consent was subsequently obtained. Selective angiography of bilateral uterine arteries revealed hypertrophic tumor-feeding vessels with intense tumor staining (**Figures 3C, D**). We performed embolization using 50-100μm pirarubicin-loaded (60 mg) microspheres until complete flow stasis was achieved. Three days following the interventional procedure, the patient initiated a comprehensive radiotherapy regimen consisting of external beam radiation therapy (50 Gy administered in 25 fractions) supplemented by intracavitary brachytherapy (30 Gy in 5 fractions of 6 Gy each).

The therapeutic response was characterized by complete cessation of vaginal bleeding within 24 hours post-intervention and substantial hematologic improvement within one week. Follow-up imaging studies performed one month after treatment completion, including MRI and PET-CT, showed complete resolution of the previously identified lesions (**Figures 3E, F**). Throughout an extended 33-month surveillance period, the patient maintained a disease-free status. Treatment-related adverse effects were limited to grade 1 toxicities (diarrhea, pyrexia, and leukopenia) as defined by CTCAE, all of which resolved spontaneously without medical intervention.

## Discussion

DEB-TACE was originally developed as a treatment for primary liver cancer, with multiple clinical studies confirming its efficacy and safety (10). In recent years, this technique has been gradually extended to other malignant tumors including non-small cell lung cancer (11, 12). A study employing gemcitabine-loaded DEB-BACE in patients with advanced NSCLC who were ineligible for standard treatment reported an objective response rate of 50.0% and a median progression-free survival of 8.0 months, along with effective alleviation of clinical symptoms such as hemoptysis and a favorable safety profile (13). Furthermore, a comparative study in advanced NSCLC patients demonstrated that DEB-BACE was associated with a superior objective response rate and a significant improvement in quality of life compared to intravenous chemotherapy (14).

In clinical practice, elderly patients with poor PS scores or underlying comorbidities often have difficulty tolerating CCRT and face poor prognoses, particularly when complicated by tumor bleeding. For this special patient population, optimal treatment options remain lacking. This study reported three cases of locally advanced solid tumors with bleeding that were ineligible for intravenous chemotherapy due to anemia or poor PS scores and showed poor response to conventional hemostatic treatments. Considering that radiotherapy might exacerbate bleeding and even become life-threatening, our multidisciplinary team innovatively adopted a combined radiotherapy and DEB-TACE approach. The results showed significant clinical efficacy in all three cases: tumor bleeding was rapidly controlled, complete clinical response was achieved after radiotherapy, and no disease progression was observed during long-term follow-up. Importantly, no grade ≥3 toxicities were observed.

From a technical standpoint, complete embolization of tumor-feeding vessels is crucial for performing DEB-TACE to manage tumor bleeding. All three patients reported in this study underwent CT angiography (CTA) prior to DEB-TACE to comprehensively evaluate tumor vascular supply. Anatomically, the vascular supply of lung cancer and cervical cancer is relatively straightforward: the former is primarily supplied by bronchial arteries (15, 16), while the latter is mainly fed by bilateral uterine arteries (17). In contrast, the vascular system of esophageal carcinoma is more complex: upper esophageal carcinoma typically receives multiple blood supplies from branches of the inferior thyroid artery (originating from the thyrocervical trunk), proximal intercostal artery branches, bronchial artery branches, and the esophageal proper artery; mid-esophageal carcinoma may receive blood supply from proximal intercostal artery branches, bronchial artery branches, the esophageal proper artery, and left gastric artery branches; while lower esophageal carcinoma is mainly supplied by the esophageal proper artery and left gastric artery branches (18). In Case 2, in addition to the aforementioned bilateral bronchial artery supply (which had been embolized with drug-eluting beads), we also identified tumor-feeding vessels from bilateral thyrocervical trunk branches, the proximal right second intercostal artery branch, the esophageal proper artery, and the relatively rare right subclavian artery branch. To avoid damaging vital organ functions, we cautiously performed selective embolization of these aberrant vessels using gelatin sponge particles. It should be particularly noted that Case 2 was a fungating-type esophageal carcinoma, whereas for ulcerative-type esophageal carcinomas, such aggressive local combination therapy may significantly increase the risk of esophageal perforation, requiring special caution in clinical practice (19). In addition to the serious complications that may arise from embolization of the blood supply to the organ harboring the tumor itself, the complex vascular anatomy of tumors and the presence of numerous collateral pathways increase the risk of non-target embolization, which may lead to severe complications in surrounding organs. DEB-TACE is contraindicated when branches supplying critical structures such as the heart, spinal cord, or brain originate from the target vessel and cannot be safely avoided during the procedure. Furthermore, the technique may also be limited in cases where key feeding arteries are too small to be selectively catheterized with currently available microcatheter systems.

Furthermore, complete embolization of tumor-feeding vessels may induce tumor tissue hypoxia. Studies in liver cancer have shown that elevated hypoxia-inducible factor-1α (HIF-1α) levels after TACE can promote angiogenesis, which may facilitate tumor metastasis/recurrence and potentially induce radioresistance (20). In our study, all three patients underwent DEB-TACE followed by radiotherapy after a 3-day interval to avoid the acute hypoxia phase (21). Nevertheless, the optimal balance between avoiding the hypoxic period and leveraging the synergistic effects of sustained chemotherapy release from drug-eluting microspheres [reported to last up to 1 month (22, 23)] and radiotherapy requires further investigation. Additionally, although all cases in this study achieved complete response following a single session of DEB-TACE combined with definitive radiotherapy, the optimal number of DEB-TACE procedures for combination therapy requires validation through larger clinical datasets.

## Conclusion

For patients with bleeding, locally advanced solid tumors who cannot tolerate CCRT, radiotherapy combined with DEB-TACE may represent a promising treatment option. However, the safety and efficacy of this technique require confirmation through larger-scale clinical studies.

## Data Availability

The original contributions presented in the study are included in the article/Supplementary Material. Further inquiries can be directed to the corresponding author.
